# A Prospective, Randomized, Controlled Comparison of Adhesive Wound Closure Devices in an Orthopaedic Patient

**DOI:** 10.5435/JAAOSGlobal-D-22-00179

**Published:** 2022-09-23

**Authors:** John F. Burke, Ian S. MacLean, J. Michael Smith, Joseph M. Hart, Mark D. Miller

**Affiliations:** From the Department of Orthopaedic Surgery, University of Virginia Medical Center, Charlottesville, VA.

## Abstract

The objective of this study was to compare Zip and Clozex with a typical closure using a running 3-0 Prolene suture.

**Methods:**

A prospective, randomized, controlled trial was conducted in patients aged 18 years and older undergoing elective orthopaedic surgical procedures between 2019 and 2021. Patients were randomized to undergo skin closure using a running 3-0 Prolene suture, Zip, or Clozex. The length and location of incision, time to close, surgeon satisfaction, and complications were recorded. The Stony Brook Scar Evaluation Scale (SBSES) was used to assess cosmesis at 2 weeks and 3 months postoperatively. Patient satisfaction and adverse events were also recorded.

**Results:**

Thirty-two patients were included in the analysis. Suture closure time was longer than Zip (266 vs 123 seconds; *P* = 0.02) and Clozex (266 vs 91 seconds; *P* = 0.005). SBSES scores were greater for Clozex at 2 weeks compared with suture (4.09 vs 2.8; *P* = 0.005). At 3 months, Clozex maintained greater scores compared with suture (3.82 vs 2.85; *P* = 0.023) and Zip (3.82 vs 3.0; *P* = 0.046).No differences were observed in patient satisfaction at any time points.

**Discussion:**

Although patient satisfaction was similar across groups, wound closure times, SBSES scores, and total cost favor Clozex compared with Prolene suture or Zip.

**ClinicalTrials.gov Registration Number:**

NCT05251064

Wound closure methods in orthopaedic surgical practices vary greatly. Although most closures typically involve a combination of braided and/or monofilament sutures in the subcutaneous fascia and fat as well as the dermal layers, the method chosen for final cutaneous closure varies widely by surgeon.^1,2^ The reasons for this are likely multifactorial. While difference in training background likely has an effect, undoubtedly conflicting reports in the literature about optimal techniques contributes greatly.^2-8^ Important aspects to consider when choosing a closure method include associated complications, cosmesis, ease of use, and time for application.^9-11^

There have been new wound closure products to reach the market such as Zip (Stryker Medical) and Clozex (Clozex Medical LLC) surgical skin closure devices purporting to increase the speed of closure, improve patient satisfaction, and decrease the rate of complications.^10-14^ Zip is a noninvasive surgical zipper with two adhesive undersides connected by several notched plastic filaments to allow coaptation and wound edge approximation. Clozex is a surgical skin closure device with multiple adhesive interlaced filaments attached to a pulling end that allows for wound edge approximation. These products are FDA-approved to promote skin apposition while reducing tension during the healing process.^15^ They are safe and effective treatment options and have been adopted in many practices.^16-18^

This study was developed to directly compare Zip, Clozex, and a typical closure method using a running 3-0 Prolene suture and to determine which provides the best cosmetic result using the Stony Brook Scar Evaluation Scale (SBSES).^1,19^ Secondary outcomes included an assessment of surgeon satisfaction with the closure method, incidence of complications, and cost of each closure method taking into consideration both the direct expense of the devices and their indirect costs (ie, time to apply and expense of surgical room utilization). The hypothesis of this study was that both surgical skin closure devices would result in faster skin closure compared with suture with equivalent cosmetic outcomes across all groups.

## Methods

A prospective, randomized, controlled trial was conducted in patients undergoing elective orthopaedic surgical procedures between 2019 and 2021. This study was reviewed and approved by the Institutional Review Board. Inclusion criteria included adults aged 18 years and older, with minimum anticipated incision length ≥3 cm, and having both willingness and ability to comply with scheduled visits and study procedures. Exclusion criteria included revision surgery, patients with chronic conditions that would compromise wound healing (eg, autoimmune disorder, chronic steroids, connective tissue disorder), or patients who were part of a vulnerable population (eg, pregnant, children, prisoner). An a priori power analysis was conducted. Based on an average SBSES score of 4.4, SD 0.73, alpha = 0.05, power = 0.08, and a minimal clinically important difference set at 1 point on the SBSES scale, there was a need to recruit 10 patients in each group to detect notable differences.^1^

Patients were enrolled and randomized to undergo final layer skin closure with a running 3-0 Prolene suture (Johnson & Johnson) with an escape stitch and tails, Zip surgical skin closure (Stryker Medical), or Clozex surgical skin closure (Clozex Medical LLC). In cases where there were multiple incisions larger than a standard portal, all incisions were closed with the same closure technique. The dermis was closed with interrupted, buried 2-0 Monocryl stitches in all groups. Fascial layers were closed at the surgeon's discretion with #0 Vicryl. The length and location of incision, time to close, and complications or issues with the closure device were recorded at the time of surgery. Surgeon satisfaction with the closure device was documented using a 10-point scale.

Wound closure device removal occurred at the 2-week follow-up appointment. The SBSES, a validated postsurgical scar evaluation score that evaluates scar appearance on a 5-point scale, including metrics such as width, height, color, suture marks, and overall appearance, was used to assess cosmetic results of skin closure at 2 weeks and 3 months postoperatively.^19^ Where multiple incisions had been closed with the same technique, these incisions were considered in aggregate during evaluation. Patient satisfaction using a 10-point scale and any adverse events were also recorded at these time points. This study was reviewed and approved by the Institutional Review Board.

All statistical analyses were conducted using SPSS statistics software. Analysis of variance tests were used to investigate differences in wound characteristics and outcome measures noted previously. Post hoc analysis with Tukey least significant differences was conducted to characterize differences among the groups, as appropriate.

## Results

Seventy-one patients were enrolled in this study. Thirty-two of these patients completed the 3-month follow-up and were included in the statistical analysis, which included 10 patients in the suture group, 11 patients in the Zip group, and 11 patients in the Clozex group. Group demographics were similar with no significant differences in age or body mass index (Table 1). All groups showed a slight female predominance (suture 60.0%, Clozex 54.5%, Zip 63.6%), and all had a similar number of smokers. There was one diabetic patient in the Zip group. No significant differences were observed in the length of incision or surgeon satisfaction with the closure method between the three groups (Table 2). All incisions were longitudinally oriented around the knee, except for one patient in the Clozex group who had an incision over the superior shoulder (Table 3). This incision was oriented along Langer lines just medial to the acromioclavicular joint for a distal clavicle resection.

**Table 1:** Patient Demographics

Factor	Clozex	Suture	Zip	ANOVA *P* Value
Age, mean ± SD	40.2 ± 18.5	40.9 ± 10.6	34.6 ± 16.7	0.606
BMI, mean ± SD	28.1 ± 5.5	30.7 ± 6.6	30.6 ± 7.9	0.704
No. of patients (female)	11 (6)	10 (6)	11 (7)	
No. of smokers	2	3	1	
No. of diabetic patients	0	0	1	

ANOVA = analysis of variance, BMI = body mass index

**Table 2:** Surgical Closure Characteristics

Factor	Clozex	Suture	Zip	ANOVA *P* Value
Length of incision (cm)	8.23 ± 4.09	9.78 ± 4.87	8.1 ± 2.78	0.601
Time to close (sec)	91.09 ± 39.53	266.31 ± 193.59	123.27 ± 124.23	0.014^a^
Surgeon satisfaction	8.18 ± 2.09	7.11 ± 3.62	8.0 ± 1.55	0.605

ANOVA = analysis of variance

a *P* < 0.05.

**Table 3:** Location of Incisions by Wound Closure Device

Factor	Clozex	Suture	Zip
Anterior knee	5	4	8
Lateral distal femur	4	1	1
Anteromedial proximal tibia	1	3	3
Medial leg	0	3	0
Anterolateral leg	0	3	0
Shoulder	1	0	0

Time to closure was markedly different among the groups. Suture closure time took more than twice as long on average compared with Zip (266 vs 123 seconds; *P* = 0.02) and nearly 3 times longer compared with Clozex (266 vs 91 seconds; *P* = 0.005) (Table 2).No difference was observed in time to closure between Clozex and Zip (*P* = 0.565). The SBSES scores were significantly greater for Clozex at 2 weeks compared with suture (4.09 vs 2.8; *P* = 0.005), with no significant differences observed between Clozex and Zip (*P* = 0.134) or suture and Zip (*P* = 0.133) (Table 4). Furthermore, at 3 months, Clozex maintained significantly greater scar evaluation scores compared with suture (3.82 vs 2.85; *P* = 0.023) and also showed significantly greater scar evaluation scores compared with Zip (3.82 vs 3.0; *P* = 0.046). No difference was observed in scar evaluation scores between Zip and suture at 3 months (*P* = 0.712). In addition, no differences were observed in patient satisfaction at 2 weeks or 3 months between the groups. When incision length was used as a covariate, these results did not change.

**Table 4:** Outcome Measures at 2 Weeks and 3 Months Postoperatively

Factor	Clozex	Suture	Zip	ANOVA *P* Value
SBSES at 2 wk	4.09 ± 0.70	2.8 ± 0.92	3.45 ± 1.21	0.018^a^
SBSES at 3 mo	3.82 ± 0.60	2.85 ± 1.20	3.0 ± 0.89	0.046^a^
Patient satisfaction at 2 wk	8.82 ± 1.33	8.33 ± 1.66	8.64 ± 1.80	0.798
Patient satisfaction at 3 mo	8.727 ± 1.73	8.11 ± 1.95	8.82 ± 1.47	0.621

ANOVA = analysis of variance, SBSES = Stony Brook Scar Evaluation Scale

a *P* < 0.05.

**Figure 1:**
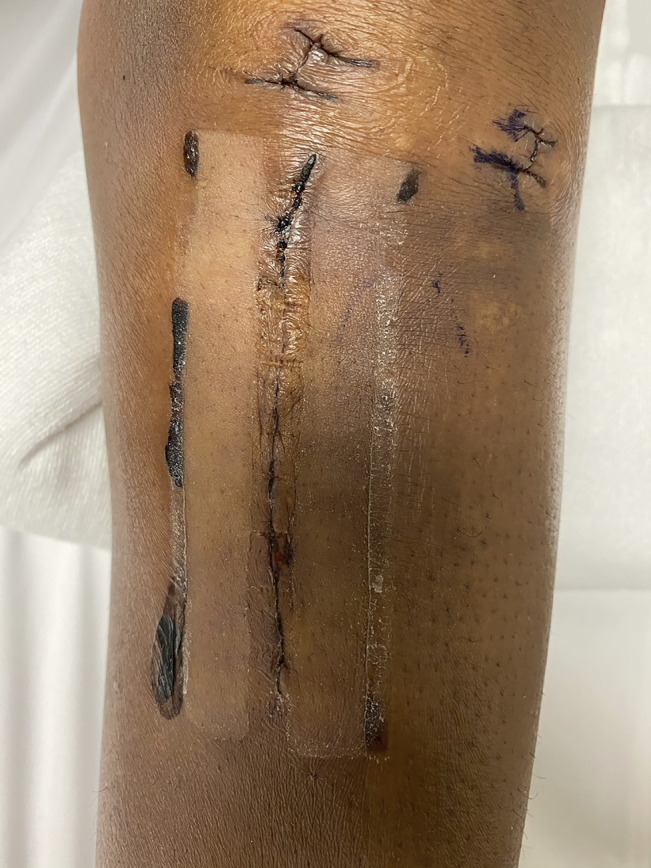
Photograph depicting blisters in a patient treated with Zip after device removal.

Regarding adverse events, one Clozex patient had mild cellulitis treated with a 10-day course of Keflex without additional complication and one Clozex patient had blisters at the 2-week follow-up that resolved without additional issue. Two Zip patients had blisters at the 2-week follow-up that resolved without additional complication (Figure 1). No adverse events were observed in the suture group at 2 weeks or 3 months.

## Discussion

This randomized, controlled trial identified better SBSES scores at 2 weeks and 3 months postoperatively when incisions were closed with Clozex as compared with Zip and a running 3-0 Prolene suture. However, patient satisfaction with the scar did not vary with the closure method. Clozex was the fastest closure method at an average of 91 seconds compared with 123 seconds for Zip and 266 seconds for suture. With a cost of $132 per minute of surgical time at our institution, the indirect time cost associated with each closure method and direct cost for each closure device are listed in Table 5.^20^ With an average incision length across the three groups of 8.65 cm, the total average costs to using Clozex, Zip, and suture are $235.40, $351.19, and $600.88, respectively.

**Table 5:** Estimated Cost of Each Closure Method

Closure Method	Unit Cost ($)	Operating Room Time Cost ($)	Total Cost ($)
Clozex	35	200.40	235.40
3-0 Prolene	15	585.88	600.88
Zip	80	271.19	351.19

Unit cost calculated based on the smallest device or a combination of devices capable of closing the study's average wound size of 8.65 cm.

Notably, two complications during application occurred in both the Clozex and Zip arms. Both issues with the Zip closure device related to difficulty getting it to adhere to the skin and required opening a second device. One issue in the Clozex arm related to adherence and necessitated the use of another device while the other related to difficulty in appropriately tensioning the device across the length of the incision. In addition, both Clozex and Zip arms had two postoperative complications noted at the 2-week follow-up visit. Three of these were due to blister formation and one, in the Clozex group, was due to mild cellulitis that resolved with a short course of oral antibiotic therapy. No long-term untoward effects were observed relating to any of these minor complications. No complications were observed relating to application or at the follow-up time points in the suture arm.

There has been an emergence of multiple surgical skin closure devices for final layer closure in the operating room, which has typically been done with suture or surgical staples. These devices include noninvasive surgical zippers and tissue adhesives, such as Zip. There have been multiple studies comparing this device with typical closure techniques. A randomized, controlled trial by Tanaka et al^21^ investigating the use of Zip in final layer closure for sternal incisions compared with a subcuticular suture found markedly shorter skin closure time and improved cosmesis based on the Vancouver Scar Scale scores for the Zip group. They reported minor complications in the Zip group including skin discoloration, epidermolysis, and exfoliation of the device, but did not find any major complications and found no difference in the incidence of wound dehiscence or infection.^21^ Zip has also been studied previously in an orthopaedic patient population undergoing total knee arthroplasty; however, these studies compared Zip with surgical staples rather than suture for final layer closure.^13,16,22,23^ In one such study, Zip was found to be favored over staples for patient-reported comfort, ease of wound care, and pain with device removal as well as superior patient and physician assessments of cosmetic outcomes.^22^ Another study also found Zip to be associated with fewer wound complications compared with staples in patients undergoing total knee arthroplasty.^16^ In addition, two biomechanical studies showed that the Zip surgical skin closure system performed superiorly to sutures and staples, holding the wound intact with reduced shear forces and higher tensile strength, reducing propensity for postsurgical scarring.^15,24^

Clozex has been noted to have a similar beneficial profile in the literature. Kuo et al^12^ conducted a randomized, controlled trial comparing Clozex with a simple running 4-0 Prolene suture among patients undergoing excision of cutaneous skin lesions. This study found that Clozex was associated with a markedly decreased time to close compared with suture and markedly improved patient and surgeon satisfaction with the cosmetic appearance of the scar 4 to 6 weeks postoperatively.^12^ Importantly, no difference was observed regarding the incidence of wound dehiscence or infection.^12^

This study provides a direct head-to-head comparison of two surgical skin closure devices currently on the market. In addition, both are compared with a cosmetic, running, subcuticular 3-0 Prolene closure. Previous studies comparing these closure devices with a running subcuticular stitch have not compared these in the setting of incisions overlying large joints. The skin over a large joint has greater mobility and experiences increased tensile forces compared with the skin over the sternum. Studies that have investigated these skin closure devices over large joints have compared them with staples, which are markedly less cosmetic because of the hatching they cause.

Overall, potential benefits of surgical skin closure devices include improved operating efficiency, reduced risk of needle stick injuries (approximately 50% of which occur with suturing needles), and improved cosmesis.^12,13,21-23,25-28^ Potential drawbacks include minor skin reaction to the adhesive used in these devices and increased cost compared with suture, which may even be offset considering increased operating efficiency and potential needle stick costs.^12,21^

There are several weaknesses to this study. First, there was only 45% follow-up with patients enrolled in this study. This study started enrolling shortly before the coronavirus disease 2019 pandemic. Because of this, a large number of patients did not return for their 2-week and/or 3-month follow-up and were excluded from the analysis. Second, clinical evaluators providing the SBSES scores at 2 weeks and 3 months were not blinded to the closure device, which may introduce bias. Third, all patients enrolled were indicated for elective orthopaedic procedures with incisions almost exclusively around the knee. Therefore, the results of this study may not be generalizable to nonelective situations or other parts of the body that experience different skin tension. In addition, on average, the incision length in the suture group was >1 cm longer than both the Zip and Clozex groups. Although this difference was not statistically significant, it may contribute to the increased closure time seen with suture. Finally, orthopaedic trainees (residents and fellows) were often responsible for wound closure. The level of training or degree of familiarity with the closure devices may have had an unanticipated influence on the outcomes (ie, time of application, satisfaction with device, complications).

In conclusion, both Clozex and Zip resulted in markedly faster closure times compared with suture. Although patient satisfaction was similar across groups, wound closure times, SBSES scores, and total cost favor Clozex. Surgeon choice in the closure method should consider multiple factors including risk profile, ease of use, cost, and cosmesis. Based on the results of this study, surgeons should consider the use of a surgical skin closure device such as Clozex for improved efficiency and cosmesis.
